# Immune adjuvant effect of *V. cholerae* O1 derived Proteoliposome coadministered by intranasal route with Vi polysaccharide from *Salmonella* Typhi

**DOI:** 10.1186/1471-2172-14-S1-S10

**Published:** 2013-02-25

**Authors:** Reinaldo Acevedo, Adriana Callicó, Yisabel Aranguren, Caridad Zayas, Yolanda Valdés, Oliver Pérez, Luis García, Valerie A Ferro, José Luis Pérez

**Affiliations:** 1 Research and Development vice-presidency of Finlay Institute, Havana, Cuba; 2 University of Strathclyde, Strathclyde Institute of Pharmacy and Biomedical Sciences, Glasgow, G4 0NR, UK

## Abstract

The proteoliposome derived from *Vibrio cholerae* O1 (PLc) is a nanoscaled structure obtained by a detergent extraction process. Intranasal (i.n) administration of PLc was immunogenic at mucosal and systemic level vs. *V. cholerae*; however the adjuvant potential of this structure for non-cholera antigens has not been proven yet. The aim of this work was to evaluate the effect of coadministering PLc with the Vi polysaccharide antigen (Poli Vi) of *S.* Typhi by the i.n route. The results showed that Poli Vi coadministered with PLc (PLc+Poli Vi) induce a higher IgA response in saliva (p<0.01) and faeces (p<0.01) than Poli Vi administered alone. Likewise, the IgG response in sera was higher in animals immunised with PLc+Poli Vi (p<0.01). Furthermore, IgG induced in sera of mice immunised with PLc+Poli Vi was similar (p>0.05) to that induced in a group of mice immunised by the parenteral route with the Cuban anti-typhoid vaccine vax-TyVi^®^, although this vaccine did not induce a mucosal response. In conclusion, this work demonstrates that PLc can be used as a mucosal adjuvant to potentiate the immune response against a polysaccharide antigen like Poli Vi.

## Background

Enteric infections induced by pathogens are one of the main causes of death all over the world [1]. *Vibrio cholerae* kills more than 100 000-130 000 persons each year, most of them children and adolescents between 5 and 19 years old. Current vaccines are mainly based on attenuated or inactivated whole bacterial cells [1]. Alternatively, our group has been working on the development of a non-living adjuvant/delivery strategy based on PLc, a proteoliposome-like structure extracted from the strain C7258 of *V. cholerae* O1 El Tor Ogawa [2]. PLc contain important antigens and immunostimulatory molecules like lipopolysaccharides (LPS), OmpU porin and MSHA. Recent work demonstrated that PLc induced a higher IgA response in mucosal fluids as well as IgG in sera against *V. cholerae* antigens; furthermore the vibriocidal activity of sera of mice immunized with PLc [2] encouraged our group to further study the potential immunoadjuvant properties of this structure. Proteoliposomes derived from bacteria have also been used as an adjuvant to potentiate the immune response against heterologous antigens [3], particularly a Neisseria derived proteosome has been used to increase the immunogenicity of LPS from *Shigella flexneri *[4]. Therefore we thought to evaluate the adjuvant potential of PLc for the capsular polysaccharide Vi of *S.* Typhi (Poli Vi). Typhoid fever caused by invasion of *S.* Typhi through enteric mucosa constitutes also a health problem; every year more than 500 000 persons die and most of them are children about 5- 19 years old from developing countries [1]. Strategies based on attenuated whole cell vaccines or purified Poli Vi vaccines have not been able to prevent Typhoid fever especially in infants [1].

## Materials and methods

**Antigens and vaccines.** PLc was obtained from the *V. cholerae* C7258 strain using a detergent protocol of extraction, reported by Pérez JL et al. [2]. The structural characterization was carried out using electron microscopy, photon correlation spectroscopy and zeta potential analysis. PLc composition was evaluated by Lowry protein assay and western blot (MSHA, LPS and OmpU) as later described [5]. Poli Vi (lot 8003) administered alone or coadministered with PLc and the vaccine vax-TyVi^®^ (lot 9009) were supplied by Finlay Institute, Havana, Cuba.

### Immunization and sample collection

Female BALB/c mice (6-8 weeks old, CENPALAB, Cuba) were immunized with a three-dose schedule, 7 days apart (0, 7, 14). Each dose of 20 μL contained 100 μg of PLc plus Poli Vi 25 μg and was administered to mice by i.n route without anesthesia (10 μL per nostril). Control groups with Poli Vi alone were administered with the same schedule by the i.n route (25 µg *per* dose *per* mouse) or one intramuscular (i.m) dose of vax-TyVi^®^ (5 µg *per* dose *per* mouse). Placebo control groups were immunized with PBS buffer. Samples were collected 7 days (saliva and faeces) or 14 days (sera) after the last dose as described elsewhere [5]. Animals were housed at the Finlay Institute animal facility. The experiment was performed by duplicate and with approval of the Finlay Institute Ethical Committee.

### Determination of antibodies by ELISA

IgG anti-Poli Vi antibodies in serum samples and IgA anti-Poli Vi antibodies in saliva and faeces were measured by indirect ELISA as described elsewhere [6]. Briefly, PoliSorp plates (Nunc, Roskilde, Denmark) were set 1 h at room temperature (20-25°C) with poli-L-Lysine at 3 μg/mL (100 μL *per* well) and then coated with Poli Vi (100 μL *per* well) at 5 μg/mL. Serum samples were diluted 1:100 and saliva and faeces 1:2 (in PBS supplemented with 1% (w/v) BSA). Peroxidase anti-mouse IgG, or anti mouse IgA were used as secondary antibodies. O-phenylene diamine (OPD) was used as the substrate to detect antibodies bound to the antigen. Optical densities were read at 492 nm using an ELISA reader (Titertek Multiskan®).

### Statistical methods

Analysis of data was carried out using ANOVA. Post test multiple comparison was performed with Graph Pad Prism 4 software (CA, USA).

## Results

### PLc extraction and characterization

**Figure 1:**
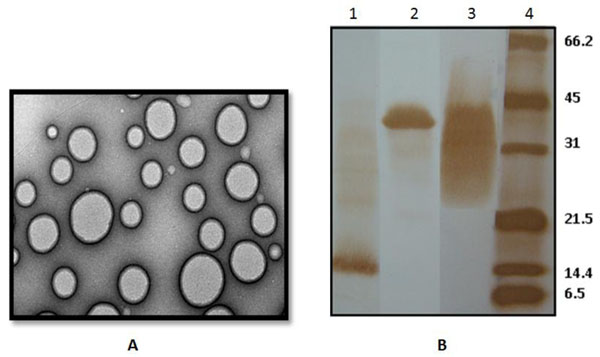
**Characterization of PLc derived from *V. cholerae* O1.** Micrograph in A was obtained by TEM and shows vesicle shape of PLc. B shows the main antigens identified in PLc by Western Blot: Lanes 1 to 3 represent blotted PLc (5 μg) and Lane 4 the biotinylated molecular weight pattern. Each Line was treated with different Mabs. (Lane 1): MAb 2F12F1 (anti MSHA), (Lane 2): MAb 9H12E6 (anti OmpU) and (Lane 3): MAb 2B4G5 (anti O-p-LPS Ogawa). Band centered for LPS is ~30 kDa (Lane 3), OmpU~38 kDa (Lane 2) and MSHA~17 kDa (Lane 1).

PLc structures were obtained from the virulent strain of C7258, *V. cholerae* O1, El Tor Ogawa using the detergent extraction protocol and observed by TEM (Figure 1A). The polydispersion index of the vesicles was 0.4 and they had a mean size of 156.9 ± 22.2 nm. Their surface charge was estimated at -23.8 ± 1.21 mV. The amount of LPS calculated by densitometric/western analysis was 0.28 ± 0.06 mg *per* 1 mg of total protein in PLc. MSHA and OmpU protein antigens, were also identified by the western blot method (Figure 1B).

### Effect of coadministration of PLc and Poli Vi

**Figure 2:**
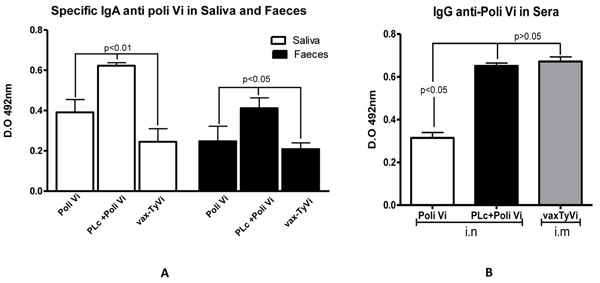
**Specific IgA (A) and IgG (B) anti Poli Vi antibody response in mice samples.** 25 μg of Poli Vi (12.5 μg each nostril) was administered with or without PLc (50 μg each nostril) in BALB/c mice (n=5) by the intranasal route (10 μL *per* nostril). A three-dose schedule (0, 7 and 14 days) was used and samples were evaluated. Vax-TyVi vaccine was used as a positive control with only one dose by the i.m. route. (A): Seven days after the last immunization, saliva (white columns) and faeces (black columns) were collected and diluted 1:2. Results are expressed as a mean of optical density units (OD) ± standard deviation. (B): Fourteen days after the last immunization, sera was obtained and diluted 1:100. Results are expressed as a mean of optical density units (OD) ± standard deviation. A placebo group was also included in each experiment and the effect was subtracted for statistical analysis. Tukey test was used to analyze the data of two experiments. P values indicate significant differences between groups.

PLc+Poli Vi coadministered by i.n route induced higher anti-Poli Vi IgA in saliva and faeces than the groups immunised with Poli Vi (i.n) alone or with vax-TyVi^®^ by i.m route (Figure 2A). Likewise, PLc+Poli Vi induced higher anti-Poli Vi IgG in sera than Poli Vi administered alone (Figure 2B). Interestingly, specific IgG anti-Poli Vi in sera induced by i.n PLc+Poli Vi formulation was comparable with that induced by one dose of vax-TyVi^®^ administered by the i.m route (Figure 2B).

## Discussion

Mucosal surfaces constitute one of the most important points of entry of infectious agents; protection against infections requires the induction of immune responses at mucosal level [7]. Polysaccharides and LPS from enteric pathogens are considered as some of the most important antigens to induce protection [8]. However, their mucosal administration has proven to be inefficient for the stimulation of the immune system [8]. Mucosal adjuvants can overcome this problem and potentiate their immune responses [9]. Protollin is a complex mix of outer membrane proteins from *Neisseria meningitidis* B and LPS from *Shig*u*ella flexneri* (proteosome), this formulation was administered by the i.n route to humans and mice and improved the immune response against LPS antigen [4,10]. Therefore, we evaluated the effect of coadministration of PLc with Poli Vi by the i.n route.

Results showed that proteoliposomes extracted from *V. cholerae* O1 (Figure 1A) contain immunogenic and immunostimulatory molecules like OmpU, MSHA and LPS (Fig 1B). Coadministration of PLc with the polysaccharide improves the mucosal and systemic immune response induced *vs.* Poli Vi (Figure 2) without affecting the immune response vs *V. cholerae* antigens (results not shown). Purified Poli Vi antigen has been used in the formulation of a parenteral vaccine against typhoid fever [11] but has never been used by a mucosal route as a *with* vaccine *purposes*. Mucosal immunization has been approached by administration by the oral route of the attenuated *S.* Typhi strain CVD 909 (HolaVax-Typhoid^®^) expressing the Vi polysaccharide [7]. In these experiments the systemic immune response was superior to that induced by the attenuated *S.* Typhi strain Ty21a which do not express Poli Vi [12]. Ty21a is also the strain used in the licensed vaccine Vivotif^®^, against typhoid fever [7]. Specific IgG anti Poli Vi in sera is crucial to avoid pathogen invasion to macrophages and evasion from immunocompetent cells [13]. The role of IgA anti Poli Vi may also be related to the blockade of pathogen invasion at a mucosal level [13], however the mechanisms of the protection induced by our formulation needs to be evaluated.

The use of mucosal vaccines has many advantages when compared to parenteral administration. Particularly, if vaccine formulations are intended against pathogens that colonize or invade via the mucosal route, like *V. cholerae* and S. Typhi [8]. Mucosal immunisation offers an attractive two-pronged approach as it stimulates both local and systemic immunity [7]. Furthermore, it also offers the potential for rapid administration in mass vaccination programs [14] without risk of injury and cross-infection through contaminated needles [15]. In addition, with a growing awareness for environmental impact, disposal of needles, decontamination costs, and incineration waste products has helped to influence development of mucosal vaccines as a greener alternative [16].

Overall, PLc has been demonstrated to be immunogenic against *V. cholerae* O1 [2] and also has immune adjuvant effects when coadministered with Poli Vi. PLc is part of the adjuvant family developed by the Finlay Institute, AFPL (Adjuvant Finlay Proteoliposome) and named AFPL2. Further studies are envisaged to evaluate the adjuvant potential of AFPL2 with other antigens and using other mucosal routes like oral to develop a multiple mucosal vaccine candidate against enteric pathogens.

## Competing interests

The authors declare that they have no competing financial interests.

## Authors' contributions

RA conceived of the study, participated in its design, discussion of results and drafted the manuscript; AC participated in the PLc production and analytical evaluation; YA participated in the evaluation of immune response; CZ participated in analytical evaluation of PLc and evaluation of immune response

YV participated in the animal work; OP participated in study design and discussion of results; LG participated in study design and discussion of results; VAF participated in discussion of results and draft of manuscript; JLP conceived of the study, participated in its design and discussion of results. All authors have read and approved the final manuscript.

## Declarations

This article has been published as part of *BMC Immunology* Volume 14 Supplement 1, 2013: Proceedings from Delivery Systems and Current strategies to drug design. The full contents of the supplement are available online at http://www.biomedcentral.com/bmcimmunol/supplements/14/S1.

## References

[B1] State of the art of new vaccine research and development http://www.who.int/vaccine_research/documents/stateoftheart/en/index.html

[B2] Pérez JL Acevedo R Callicó A Fernández Y Cedré B Año G González L Falero G Talavera A Pérez O A proteoliposome based formulation administered by the nasal route produces vibriocidal antibodies against El Tor Ogawa *Vibrio cholerae* O1 in balb/c mice Vaccine 2009 25 205 212 18996426 10.1016/j.vaccine.2008.10.052

[B3] Pérez O Lastre M Cabrera O del Campo J Bracho G Cuello M Acevedo R New Vaccines Require Potent Adjuvants like AFPL1 and AFCo1 Scandinavian Journal of Immunology 2007 66 271 277 10.1111/j.1365-3083.2007.01981.x 17635804

[B4] Orr N Arnon R Rubin G Cohen D Bercovier H Lowell GH Enhancement of anti-Shigella lipopolysaccharide (LPS) response by addition of the cholera toxin B subunit to oral and intranasal proteosome-Shigella flexneri 2a LPS vaccines Infect Immun 1994 62 11 5198 5200 7927807 10.1128/iai.62.11.5198-5200.1994 PMC303249

[B5] Acevedo R Callicó A del Campo J González E Cedré B González L Romeu B Zayas C Lastre M Fernández S Intranasal administration of proteoliposome-derived cochleates from Vibrio cholerae O1 induce mucosal and systemic immune responses in mice Methods 2009 49 4 309 315 10.1016/j.ymeth.2009.03.027 19545630

[B6] Ramirez JC Serrano B Lara M Farinas M Mirabal M Sifontes S Garcia I Gonzales P Lopes Y Garcia A Study of immunogenicity in mice of the Cuban Vi Polysaccharide Typhoid vaccine, vax-TyVi VacciMonitor 2006 15 2 1 4

[B7] Yuki Y Kiyono H Mucosal Vaccines: novel advances in technology and delivery Expert Rev Vaccines 2009 8 8 1083 1097 10.1586/erv.09.61 19627189

[B8] Dietrich G Griot-Wenk M Metcalfe IC Lang AB Viret JF Experience with registered mucosal vaccines Vaccine 2003 21 678 683 10.1016/S0264-410X(02)00579-0 12531339

[B9] Leroux-Roels G Unmet needs in modern vaccinology: adjuvants to improve the immune response Vaccine 2010 28S 25 36 10.1016/j.vaccine.2010.07.021 20713254

[B10] Fries LF Montemarano AD Mallett CP Taylor DN Hale TL Lowell GH Safety and immunogenicity of a proteosome-Shigella Flexneri 2a lipopolysaccharide vaccine administered intranasally to healthy adults Infect Immun 2001 69 7 4545 4553 10.1128/IAI.69.7.4545-4553.2001 11401998 PMC98531

[B11] Guzman CA Borsutzky S Griot-Wenk M Metcalfe IC Pearman J Collioud A Favre D D G Vaccines against typhoid fever Vaccine 2006 24 3804 3811 10.1016/j.vaccine.2005.07.111 16278037

[B12] Garmory HS Brown KA Titball RW Salmonella vaccines for use in humans: present and future perspectives FEMS Microbiology Reviews 2002 26 339 353 12413664 10.1111/j.1574-6976.2002.tb00619.x

[B13] Raffatellu M Chessa D Wilson RP Tukel C Akcelik M Baumler AJ Capsule-Mediated Immune Evasion: a New Hypothesis Explaining Aspects of Typhoid Fever Pathogenesis Infect Immu 2006 74 1 19 27 10.1128/IAI.74.1.19-27.2006 PMC1346610 16368953

[B14] Giudice EL Campbell JD Needle-free vaccine delivery Adv Drug Deliver Rev 2006 58 1 68 89 10.1016/j.addr.2005.12.003 16564111

[B15] Brody S Declining HIV rates in Uganda: due to cleaner needles, not abstinence or condoms Int J STD AIDS 2004 15 440 441 10.1258/0956462041211324 15228727

[B16] Report of the third meeting of the Steering Committee on Immunization Safety http://www.who.int/vaccines-documents/DocsPDF03/www746.pdf 12478997

